# Self-assembly of toroidal proteins explored using native mass spectrometry†
†Electronic supplementary information (ESI) available. See DOI: 10.1039/c8sc01379a


**DOI:** 10.1039/c8sc01379a

**Published:** 2018-06-18

**Authors:** N. Amy Yewdall, Timothy M. Allison, F. Grant Pearce, Carol V. Robinson, Juliet A. Gerrard

**Affiliations:** a School of Biological Sciences , School of Chemical Sciences , University of Auckland , Auckland 1010 , New Zealand; b Biomolecular Interaction Centre , School of Biological Sciences , University of Canterbury , Christchurch 8140 , New Zealand; c Department of Chemistry , University of Oxford , Oxford OX1 5QY , UK; d MacDiarmid Institute for Advanced Materials and Nanotechnology , Victoria University , Wellington 6140 , New Zealand

## Abstract

The peroxiredoxins are a well characterised family of toroidal proteins which can self-assemble into a striking array of quaternary structures, including protein nanotubes, making them attractive as building blocks for nanotechnology. Tools to characterise these assemblies are currently scarce. Here, assemblies of peroxiredoxin proteins were examined using native mass spectrometry and complementary solution techniques. We demonstrated unequivocally that tube formation is fully reversible, a useful feature in a molecular switch. Simple assembly of individual toroids was shown to be tunable by pH and the presence of a histidine tag. Collision induced dissociation experiments on peroxiredoxin rings revealed a highly unusual symmetrical disassembly pathway, consistent with the structure disassembling as a hexamer of dimers. This study provides the foundation for the rational design and precise characterisation of peroxiredoxin protein structures where self-assembly can be harnessed as a key feature for applications in nanotechnology.

## Abstract 2

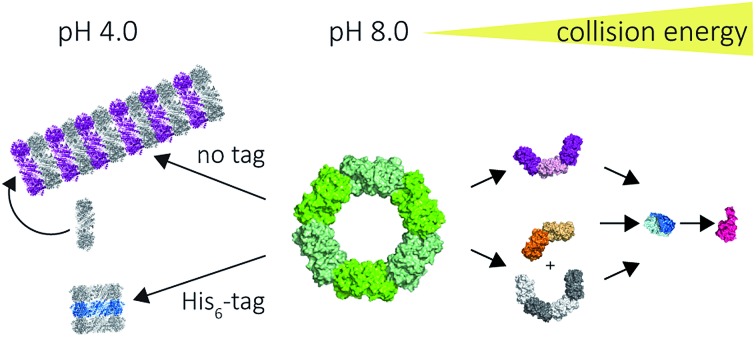
The peroxiredoxins are a well characterised family of toroidal proteins which can self-assemble into a striking array of quaternary structures, including protein nanotubes, making them attractive as building blocks for nanotechnology.

## Introduction

**Fig. 1:**
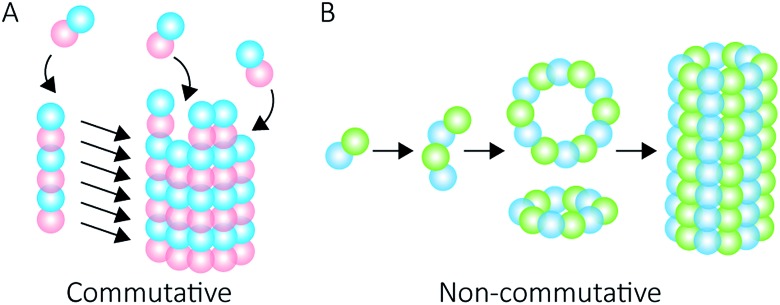
Self-assembly mechanisms. Commutative assembly (A) occurs when steps can be interchanged along an overall pathway that leads to a final “open” structure. Non-commutative assembly (B) involves the progressive formation of a final structure through building up from a defined set of intermediates.

Proteins intrinsically self-assemble into diverse three dimensional structures within the nanoscale, making them viable building blocks for applications in nanotechnology. The self-assembly of supramolecular structures can occur *via* two mechanisms: commutatively, where multiple pathways can lead to a final structure, and non-commutatively, where a hierarchical assembly occurs towards the formation of a final structure^1^,^2^ (Fig. 1). Understanding the non-covalent self-assembly mechanism of these materials is fundamental for their application. However, studying the pathways of assembly is not straightforward, and often relies on modelling techniques.^5^ Native mass spectrometry (MS) is a sensitive method that can be used to examine protein complexes^6^–^10^ and, in this particular case, unravel the intermediate pathways by which the protein self-assembled structures arise.

**Fig. 2:**
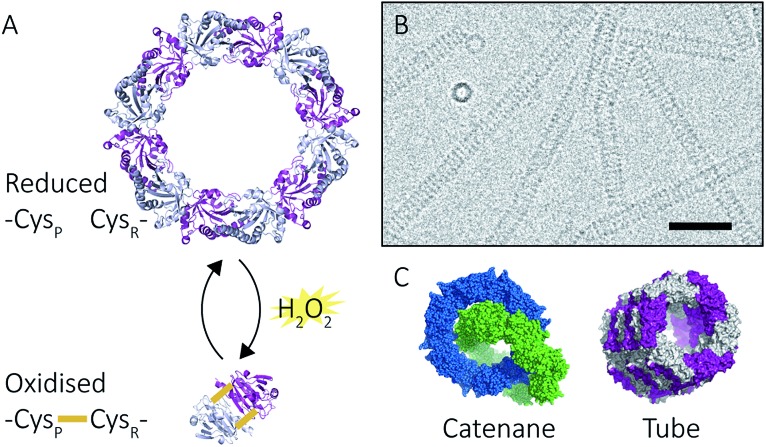
Peroxiredoxin structural diversity. Human peroxiredoxin 3 has a reversible redox-sensitive switch which modulates its structure (A) between a dodecameric ring and a dimer. Human peroxiredoxin 3 also forms long protein tubes at pH 4.0 (B) as observed using cryo-EM (image courtesy of Hari Venugopal). Scale bar is 50 nm. Crystal structures of high molecular weight forms of peroxiredoxin (C) as catenanes (left, PDB: 1ZYE)^3^ and protein tubes (right, PDB: ; 5JCG).^4^

Peroxiredoxins are an ideal model system for the study of protein supramolecular self-assembly as a subgroup of this protein family (2-Cys peroxiredoxins‡
‡The superfamily can be split into three classes depending on their enzymatic mechanism: typical two cysteine (2-Cys) peroxiredoxin, atypical 2-Cys peroxiredoxin and one cysteine (1-Cys) peroxiredoxin.
^,^^11^) can exhibit an assortment of structures (Fig. 2) that include: dimers, rings composed of 10 or 12 subunits, catenanes,^3^ cages,^12^ spherical protein clusters^13^ and protein nanotubes.^4^,^14^–^16^ Solution conditions have a profound effect on the equilibrium of these structures,^15^–^17^ and it is the prospect of controlling the formation of particular nanoscale architectures that renders peroxiredoxins attractive building blocks for nanotechnological applications.^18^–^20^ Human peroxiredoxin 3 proteins exhibit many of the aforementioned nanostructures (Fig. 2) and, like other typical 2-Cys peroxiredoxins, possess a well-documented redox-sensitive structural switch between oxidised dimeric species and reduced, ringed dodecameric species composed of six dimers^15^,^21^–^23^ (Fig. 2A). This structural shift hinges on the positioning of the active site, which is in turn influenced by the redox state of an active-site cysteine.

In addition to dimers and rings, human peroxiredoxin 3 self-assembles into high molecular weight (HMW) protein tubes of hundreds of nanometres in length when dialysed into pH 4.0 buffer over 20 hours 15,17 (Fig. 2B). This makes peroxiredoxin assembly an excellent model with which to develop mass spectrometric methods for observing self-assembly mechanisms.

In contrast to the redox-induced structural switch, the mechanism by which the HMW species assemble, although assumed to be non-commutative,^2^ has yet to be demonstrated. Inspection of the HMW structures reveals the protein secondary structure undergoes no notable conformation changes concomitant with the assembly of protein nanotubes.^4^ Instead, it was postulated^4^ that lowered solution pH increases the positive charge of a surface histidine residue that enhances favourable electrostatic and hydrogen-bonding interactions at the protein–protein interface and that the stacking is reversible.^15^,^17^

The formation of certain nanostructures can also be programmed into the amino acid sequence of peroxiredoxins.^23^,^24^ Histidine tags located at the protein N-terminus (His_6_-tag) both stabilise peroxiredoxin rings,^25^ as well as alter the transition pH at which self-assembly into HMW protein tubes occurs.^26^ The peroxiredoxin N-termini project into the inner cavity of the ring, and His_6_-tags have been used for binding useful moieties,^18^ and for binding to surfaces.^26^ The presence or absence of the histidine tag thus facilitates a tuneable system by which protein supramolecular self-assembly complexes can be designed, which could be applied to other self-assembling protein systems.§
§Cleaved refers to protein with the N-terminal His_6_-tag removed using TEV protease cleavage. These proteins retain five additional residues (GIDFT) at the N-terminus before the wildtype human peroxiredoxin 3 sequence.


In this study, native MS, analytical ultracentrifugation (AUC) and size exclusion chromatography coupled with static light scattering (SEC-SLS) were used to probe supramolecular self-assembly for both cleaved and His_6_-tagged human peroxiredoxin 3. HMW protein nanotubes were demonstrated to unequivocally form *via* a non-commutative mechanism in a sub-minute timescale, with the His_6_-tag hindering the formation of long tubes at pH 4.0. The reversibility of HMW tube formation was also verified. The associations involved in peroxiredoxin ring assembly were examined using collision-induced dissociation (CID) experiments^27^ that suggest the presence of a weak dimer–dimer interface. These insights augment our understanding of peroxiredoxin protein ring assembly, which is useful for nanotechnology applications and for understanding protein self-assembly at a fundamental level.

## Results & discussion

### Protein nanotubes form non-commutatively at pH 4.0

**Fig. 3:**
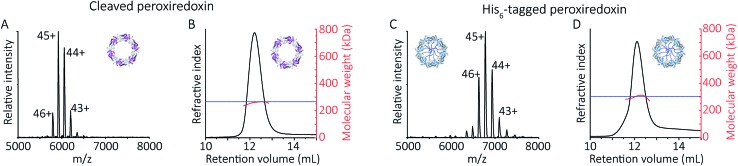
Native MS and SEC-SLS data of peroxiredoxin proteins in 100 mM ammonium acetate, pH 8.0. 20 μM of cleaved protein (A) and 20 μM His_6_-tagged protein (C) in 100 mM ammonium acetate, pH 8.0. Mass spectra peaks were assigned charged states from 43+ to 46+. In both cases, the spectra demonstrate the proteins are dodecameric oligomers, with experimental MWs of 266 560 ± 68 Da and 305 045 ± 89 Da for cleaved and His_6_-tagged variants respectively. The trap collision energy used to record both spectra was 20 V. SEC-SLS of 20 μM cleaved protein (B) and 20 μM His_6_-tagged protein (D), both in 100 mM ammonium acetate, pH 8.0. The refractive index (black) and right-angle light scattering were used to calculate MWs (red) of particles in solution. The main protein peak is a dodecamer (∼266 kDa for cleaved and ∼300 kDa for tagged), which agree with the theoretical MW for each species (blue line).

To follow the low-pH driven assembly behaviour of peroxiredoxin nanotubes, we first measured the oligomeric state of peroxiredoxin 3 at pH 8.0 using native MS. Both cleaved and His_6_-tagged proteins were buffer exchanged into 100 mM ammonium acetate (pH 8.0) and mass spectra recorded (Fig. 3A and C). Both peroxiredoxin constructs were dodecamers, consistent with size exclusion chromatography (Fig. 3B and D, and S1†) as well as previous reports of dodecameric peroxiredoxin structure.^4^,^15^,^17^ Notably, for both proteins at pH 8.0, no other stoichiometries were observed, confirming a homogeneous single population of oligomeric state in solution.

**Fig. 4:**
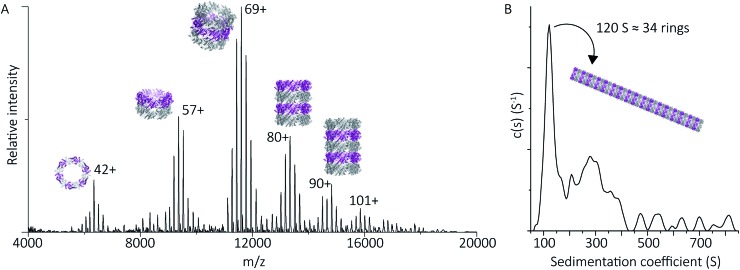
Peroxiredoxins associate into HMW stacks of rings at pH 4.0. The mass spectrum of cleaved human peroxiredoxin 3 (A) has distinct charge state series for each discrete HMW stacked species, with the MWs (Table S1†) corresponding to stacks of one to six rings. The highest intensity charge states in each series are labelled. Monomer and dimer species were not observed. AUC (B) shows a population of large species, with a main population at 120 S that corresponds to 34 stacked rings at 9 MDa.

To probe directly the mechanism of pH-controlled peroxiredoxin protein tube assembly, the proteins were buffer exchanged in 100 mM ammonium acetate from pH 8.0 to 4.0 and analysed by native MS. HMW species for cleaved peroxiredoxin, with masses corresponding to between one and seven rings, were immediately observed upon initiation of electrospray with discrete charge-state series observed for each stack of protein toroids (Fig. 4A) previously observed only by TEM and cryo-EM.^15^,^17^ Consistent with the observations in the mass spectra at pH 8.0, intermediate species composed of partial rings, such as 4-mers, 6-mers or 8-mers, were not detected as the stacked ring stoichiometry evolves, indicating that the mechanism of HMW protein tube formation occurs non-commutatively, and exclusively through the association of rings. Intriguingly, the most populated species was a stack of three rings, a structural unit observed in the X-ray crystal structure.^4^

HMW tube assemblies were also observed by analytical ultracentrifugation (AUC) (Fig. 4B). The size of the cleaved peroxiredoxin HMW species, observed by AUC, were larger than those recorded by native MS with a dominating peak at c(s) of 120 S, corresponding to a species with a mass of approximately 9 MDa (>30 rings) consistent with a population of protein tubes of heterogeneous length. The difference in length likely reflects the different timescales and conditions of the two experiments.

### N-Terminal His_6_-tag influences the self-assembly of HMW protein tubes at pH 4.0

**Fig. 5:**
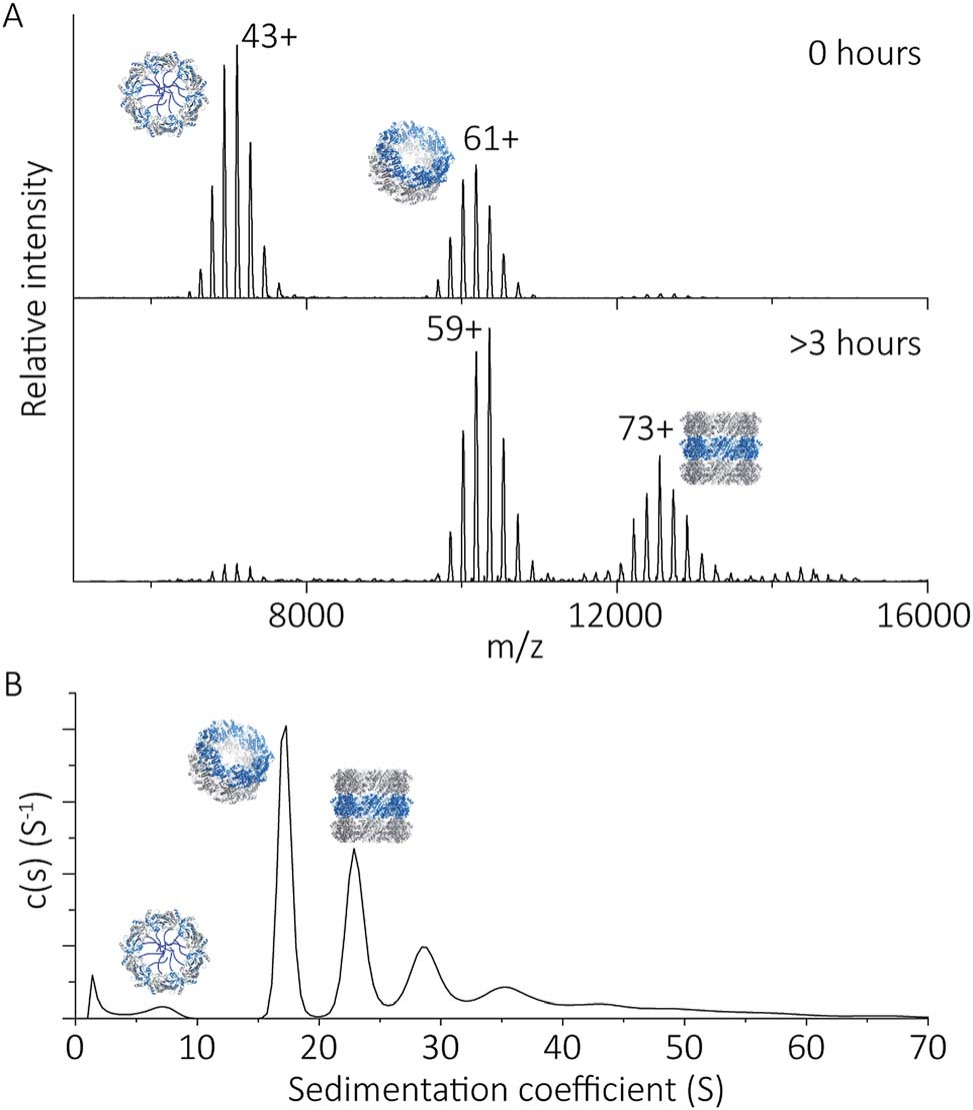
His_6_-tagged proteins form shorter tubes at pH 4.0. Mass spectra (A) comparing the assembly of peroxiredoxin rings at 0 hours after the time of buffer exchange and at 3 hours at pH 4.0. Over this time period, the population of stacked rings shifts to that of higher MW. Charged states are labelled for the most intense peak. The masses derived from this spectrum are located in Table S2.† AUC analysis (B) shows similar stacking behaviour for His_6_-tagged protein, with sedimentation coefficients for the three highest peaks of 17 S, 23 S, 29 S. These correspond to the formation of HMW species composed of two to four rings.

To explore the potential of the histidine tag to control self-assembly, we compared the peroxiredoxin with and without the tag. Whilst the cleaved protein was observed by native MS to form tubes composed of 1–7 rings, His_6_-tagged peroxiredoxin at pH 4.0 forms shorter tubes composed of 2 to 3 rings (Fig. 5A). For this protein, the ring stacking phenomenon leading to long tubes appeared to occur more slowly, as similar species are observed by both AUC and native MS collected 3 hours after buffer exchange (Fig. 5B).

His_6_-tagged peroxiredoxin has an extra 27 amino acids protruding from the N-terminus of the protein. These flexible extensions are situated in the centre of the ring, and may sterically hinder the formation of contacts at the ring–ring interface at this pH. This shifts the equilibrium of ring association towards that of single rings, disfavouring the formation of larger structures, consistent with our observations. The differences in assembly between the cleaved and His_6_-tagged forms confirm that modification of the protein in this way is a method which can be employed to control self-assembly.

### The reversible self-assembly of protein tubes

**Fig. 6:**
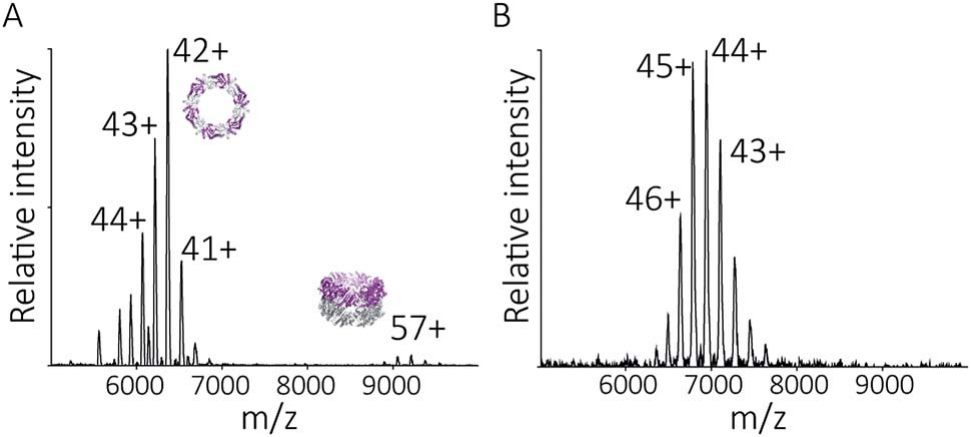
The pH-switchable HMW protein tube formation is reversible. Peroxiredoxin proteins were buffer exchanged from 100 mM ammonium acetate, pH 4.0, back into 100 mM ammonium acetate, pH 8.0, prior to injection into the MS instrument. The spectra for cleaved protein (A) shows the majority of the species returned to single rings of an experimental mass of 267 346 ± 93 Da. A very small population of two stacked rings (around 9000 *m*/*z*) can also be detected. The spectra for His_6_-tagged protein (B) shows only a single population of single rings with an experimental MW of 305 164 ± 80 Da.

Given we could use native MS to detect discrete dodecameric protein species at pH 8.0, and the formation of HMW species at pH 4.0, we were able to observe whether protein tube self-assembly was reversible when solution pH is reverted back to 8.0. The pH of 100 mM ammonium acetate solutions containing peroxiredoxin proteins was increased from pH 4.0 to pH 8.0, and mass spectra show that disassembly of the stacked rings back into predominantly single rings occurs. There were no differences in the disassembly for both cleaved and His_6_-tagged proteins (Fig. 6), and much like low-pH induced tube formation, occurs in under 60 seconds. This is the first time this switch has been directly observed in peroxiredoxins, suggesting the protein is capable of undergoing adaptive cycles of assembly and disassembly responding to changes in solution pH. This dynamic self-assembly is a highly desirable feature for a building block for use in nanotechnological applications.

### Peroxiredoxin rings dissociate in a non-classical manner as gas-phase collision energy is increased

Protein quaternary structures and interface strengths can be further interrogated by intentionally disrupting the gas phase oligomers by increasing the protein ion energy, and recording the pathways by which protein dissociation proceeds. Ion energy is increased by application of collisional activation, where the protein ions are collided with inert gas molecules at different levels of accelerating potential in the mass spectrometer. Collisional activation causes oligomeric proteins to dissociate, or fall apart, into constituent monomers or higher-order sub-oligomers. The pathway of protein dissociation, that is, what subunits dissociate and at what level of collisional activation, is directly related to the strengths of the subunit interfaces.

**Fig. 7:**
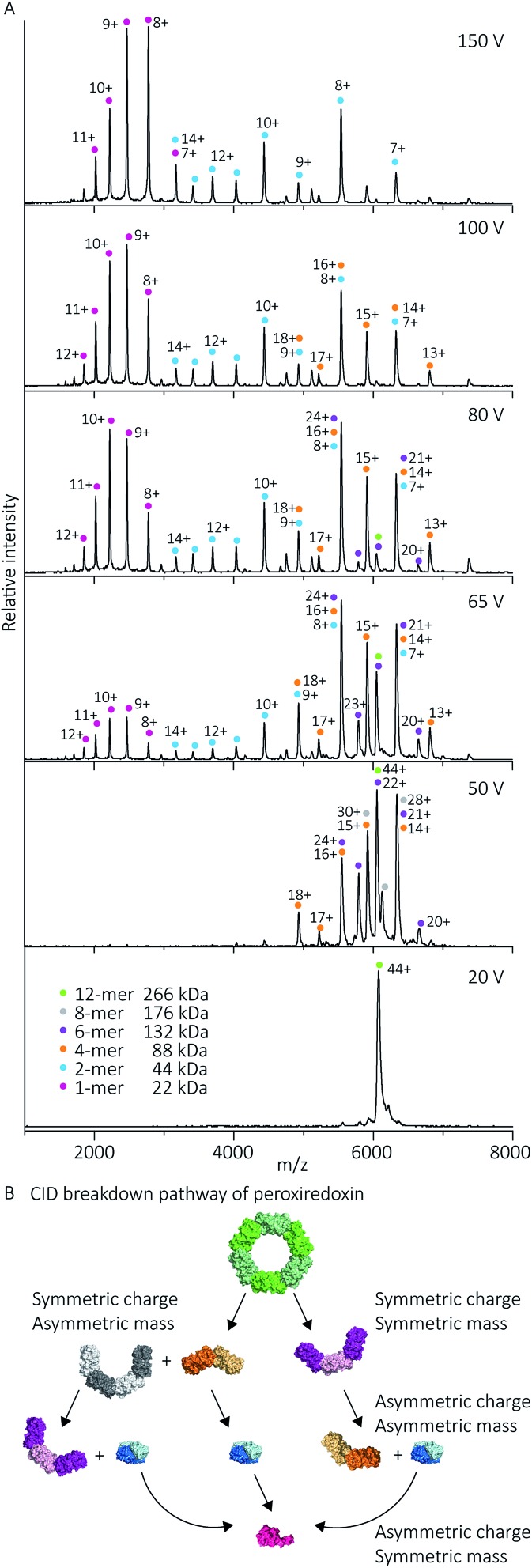
Cleaved peroxiredoxin CID pathway. MS/MS spectra (A) recorded at different trap voltages (indicated on right) follows the dissociation of the isolated 6080 *m*/*z* (44+) species of cleaved protein. Peaks were assigned to each charge state series of the oligomer as indicated in the legend. The schematic (B) illustrates a putative pathway of peroxiredoxin ring dissociation as trap voltage is increased. The colours of each species corresponds to the above spectra legend. Peroxiredoxin proteins predominantly dissociate in a non-classical manner with an array of distributions. Symmetric charge and symmetric mass dissociations (12-mer → 6-mer) dominate the lower collision energy (50 V) with a small proportion of symmetric charge and asymmetric mass dissociation also observed (12-mer → 8-mer + 4-mer). At higher collision energy, asymmetric charge and mass dissociation occurs, with dimeric subunits unfolding and dissociating. This unusual dissociation pattern is followed by further disruption of the dimer into monomers, which is more similar to classical CID. Similar dissociation patterns were also observed for His_6_-tagged protein (Fig. S3†).

Using collision-induced dissociation (CID) we probed the stability of the peroxiredoxin protein rings at pH 8.0 to gain knowledge about the strength of their internal interfaces. Tandem MS was first performed on cleaved peroxiredoxin, selecting the species at *m*/*z* 6080 (44+) (Fig. 7A), prior to collisional activation. The CID pathway observed starts with the initial disruption of the peroxiredoxin dodecamer into 6-mers, or 4-mers and 8-mers (Fig. 7B). The majority of the lower energy dissociation processes are a mixture of dissociation that is symmetric and asymmetric by mass, with all symmetric by charge (Fig. 7B).

This dissociation of the cleaved peroxiredoxin rings in a symmetric by mass manner is in contrast to the usual pathway of protein dissociation in the gas phase (Fig. 7A). The usual pathway, for example the GroEL tetradecamer which has been well characterised by native MS,^28^–^32^ involves the ejection of a highly charged monomer bearing a large proportion of the total charge.^27^,^33^ In these cases, gas phase protein unfolding redistributes charge before subunit ejection. In contrast, the symmetric charge distribution on the peroxiredoxin daughter species suggests that the cleaved peroxiredoxin structure acts as a hexamer of dimers, with weaker interactions between, rather than within, the dimers driving the disassembly pathway. That is, the energy of unfolding subunits is greater than the energy required to disrupt the dimer–dimer interface of the dodecamers. This symmetric dissociation pathway has been observed in experiments employing collision-induced dissociation,^34^ surface-induced dissociation,^35^,^36^ and ultra-violet photodissociation.^37^ Dissociation *via* these symmetric pathways is usually related to structural features of the protein, such as differing protein–protein interface sizes.^33^ Consistent with this, the unusual dissociation pathway observed for human peroxiredoxin 3 can be rationalised by analysis of the interactions at the subunit interfaces. The dimer–dimer interface has a surface area of 656 Å^2^, which is three times smaller than for the monomer–monomer interface that forms the dimer (1977 Å^2^). The interactions at the larger monomer–monomer interface involve not only the formation of an extended anti-parallel β-sheet between the two monomers, but also the intimate contacts of the C-terminus α-helix which embraces the adjacent monomer.^4^

Whilst the majority of the observed dissociation is symmetric by charge, the final decomposition of dimer to monomer resembles classical CID,^27^ where subunit unfolding prompts asymmetric charge dissociation to occur (Fig. 7a). At the highest dissociation energies sampled, monomeric species with charge state distributions suggestive of asymmetric dissociation processes dominate the relative abundance of oligomers. By piecing all this information together, we can generate a complete description of the collision-induced dissociation pathway (Fig. 7B), which is consistent with the structure of human peroxiredoxin 3.

Compact dimeric protein structures containing intra-monomer cross links, such as disulfide bonds, tend to dissociate with symmetric charge distribution.^38^,^39^ To ensure the presence of disulfide bonds were not influencing the dissociation pathway, samples used for MS were analysed by non-reducing SDS-PAGE (Fig. S2†). The lack of disulfide-bonded dimers supports the observation, using MS, of weak dimer–dimer interface strength causing the dissociation of dodecamers into dimers (or equivalent substructures).

Given that the His_6_-tag influences peroxiredoxin self-assembly, its dissociation pathway was also probed using CID. Similar symmetric dissociation patterns were also observed for His_6_-tagged protein (Fig. S3†), which is consistent with the flexible His_6_-tags not contributing to the protein–protein interface strength.

These gas phase dissociation behaviours suggest that, in contrast to the HMW tube formation mechanism, peroxiredoxin rings can assemble *via* a commutative pathway through potential intermediate states not normally observable kinetically. Destabilised peroxiredoxin rings were reported to form a small population of these intermediates by AUC.^23^ This modular assembly of peroxiredoxins facilitates these proteins to access unique architectures such as catenanes.^3^

## Conclusions

**Fig. 8:**
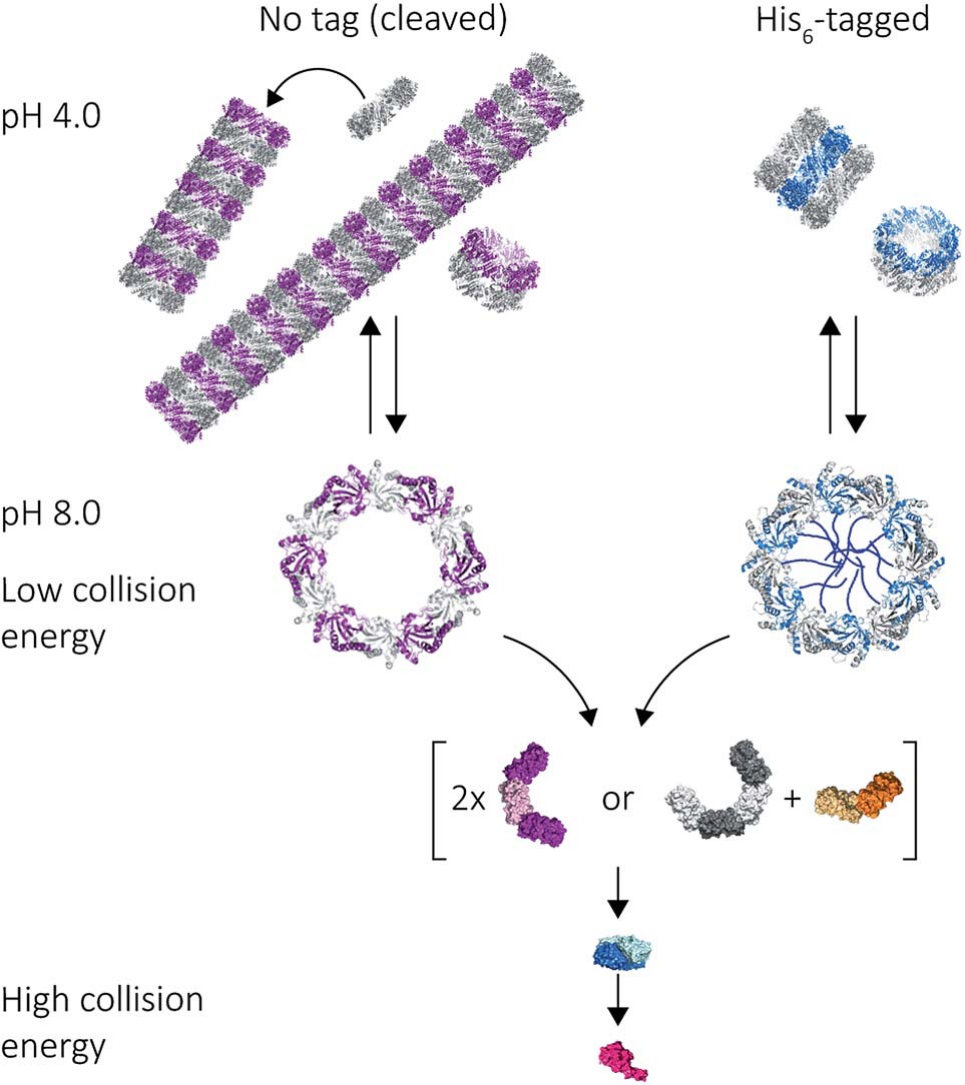
Mass spectrometry used to probe various stages of human peroxiredoxin 3 protein self-assembly. Peroxiredoxin toroids can self-assemble into protein tubes at lowered pH, with lengths tuneable using an N-terminal histidine tag. By altering collision activation energy, protein rings were disrupted into constituent intermediate species of 6-mers, 8-mers and 4-mers; all of which further dissociate symmetrically *via* the ejection of dimers.

Our results on the self-assembly of peroxiredoxin demonstrate the versatility of native MS for monitoring controlled assembly of protein complexes (Fig. 8). Peroxiredoxin can be designed to assemble into different length tubes by changing the pH in the presence of, or in the absence of, a simple histidine tag. This study highlights the power of mass spectrometry to probe not only how small changes in protein sequence control the size of the assembled protein complex, but also the mechanism of assembly. Peroxiredoxins assemble in a non-commutative manner, where units associate hierarchically, forming first dimers into toroids, then stacks of rings, which constitute the protein tubes. This process was demonstrated to be reversible. The N-terminal His_6_-tag afforded shorter tubes.

An unusual dissociation pathway for peroxiredoxin protein rings was observed as they undergo CID. This non-classical pathway is useful for understanding the self-assembly of peroxiredoxin rings: where dimers can associate as intermediate 4-mers, 6-mers and 8-mers. The dissociation of entire dimers, as opposed to monomers, as collision energy is increased is indicative and consistent with predicted interface strengths and will be important for characterisation of functionalised structures.

Together, these results attest to the multifaceted appeal of using peroxiredoxin proteins, both cleaved and His_6_-tagged, not only as a model for studying toroidal protein supramolecular assembly, but also as building blocks for applications in nanotechnology. The hierarchical and reversible association of these proteins will mean each step of assembly is tailorable for the creation of new smart materials. It also enables the study of the protein nanotubes in molecular detail, providing scope for characterisation of large scaffolds where precise positioning of functional moieties is critical,^40^ but for which tools are not yet available.

## Experimental

### Materials and protein preparation

Unless otherwise stated, all chemicals were purchased from Sigma-Aldrich (Auckland, New Zealand). Recombinant human peroxiredoxin 3 protein with an N-terminal histidine tag was expressed and purified as previously reported.^4^,^15^ For cleaved samples, the histidine tag was removed as described.^4^ Protein samples were stored at 10 mg mL^–1^ in 20 mM HEPES pH 8.0, 150 mM NaCl, 2 mM tris(2-carboxyethyl)phosphine, 5% (v/v) glycerol at –80 °C. Prior to all experiments, proteins were thawed and buffer exchanged using Bio-Spin 6 columns (BioRad). Protein concentrations were measured at *A*_280_ using an extinction coefficient of 0.9 on a NanoDrop spectrophotometer.

### Native mass spectrometry

The protein was buffer exchanged into 100 mM ammonium acetate, pH 8.0 or pH 4.0, then diluted to 20 μM. Rapid dilution (of 1 in 20 for final concentration of 20 μM monomeric concentration) of concentrated protein at pH 8.0 to pH 4.0 ammonium acetate was also performed, with similar results to using buffer exchange columns. Nanoflow electrospray ionization mass spectrometry was performed on a Synapt HDMS quadrupole time-of-flight mass spectrometer (Micromass UK Ltd, Waters Corporation, Manchester, United Kingdom). 5 μL protein solution (monomer concentration of 20 μM) was introduced to the spectrometer using gold-coated borosilicate capillaries prepared in-house as previously described.^41^

Experiments were performed under positive ion mode, with the following instrument parameters: capillary voltage 1.35 kV; sample cone voltage 15–30 V; extraction cone voltage 1–5 V; source temperature 20 °C; ion trap collision energy 10–20 V; transfer collision energy 4–5 V; trap flow 3.5 mL min^–1^ (argon gas); ion mobility source 20–40 mL min^–1^ (argon gas); backing pressure 5.0 mBar (nitrogen gas).

Tandem mass spectrometry was performed, where the species (*m*/*z* 6080 for cleaved protein and *m*/*z* 6770 for His_6_-tagged protein) were quadrupole isolated. The collision energy was increased in a stepwise manner using trap CE voltage on the instrument to give a collection of spectra.

The time from initiation of buffer exchange to recording of mass spectra was 60 seconds, and spectra recorded after 3 hours showed no change in the populations of oligomeric states. The rapid formation of the cleaved peroxiredoxin HMW tubes excluded the possibility of determining kinetic parameters for assembly.

Spectra were analysed using MassLynx software (Waters) and UniDec.^42^ The raw spectra were smoothed and background subtracted.

### Analytical ultracentrifugation (AUC)

Sedimentation velocity experiments were executed using the Beckman coulter model XLI analytical centrifuge with UV-visible scanning optics. 400 μL buffer reference (100 mM ammonium acetate at either pH 8.0 or pH 4.0) solution and 380 μL protein sample solution (20 μM monomer concentration) were loaded into 12 mm double sector cells with quartz windows and mounted into the An-60 Ti eight-hole rotor. The various rotor speeds and wavelengths used for each sample are listed on Table S3.† Radial absorbance data were collected at a single wavelength without averaging, using a 0.003 cm step size for a total of at least 70 scans. All data were collected at 20 °C. *SEDNTERP* was used to calculate the partial specific volume of both His_6_-tagged and cleaved protein (0.7405 g mL^–1^ and 0.7423 g mL^–1^, respectively), the solvent density (1.006 g mL^–1^) and viscosity (0.01031 poise).^43^ Data were fitted to a continuous c(s) distribution model at a resolution of 300 and a confidence level of 0.95 using *SEDFIT*.^44^

### Size exclusion chromatography coupled with static light scattering (SEC-SLS)

A Superdex 200 Increase 10/300 GL (GE Healthcare) was connected to a Viscotek 302-040 Triple Detector GPC/SEC system (ATA Scientific) operated at 28 °C and equilibrated with the said buffer. 100 μL protein samples were injected into the equilibrated column and were eluted at a flow rate of 0.5 mL min^–1^. The absolute molecular weight was calculated from the refractive index and right-angle light scattering measurements calibrated against bovine serum albumin (BSA) (66.5 kDa). BSA was run on either side of the protein sample sequence to ensure consistency throughout the sample sequence in experiments which would take a few hours. Calibration and calculations of absolute molecular weights were done on OmniSEC (Malvern Company).

For additional methods (SDS-PAGE gel and assessment of interface surface area), please see ESI.†

## Conflicts of interest

There are no conflicts to declare.

## Supplementary Material

Supplementary information
