# Supplementary material for: Self-assembly of toroidal proteins explored using native mass spectrometry
Source: Chem Sci. 2018 Jun 18;9(28):6099–106. doi: 10.1039/c8sc01379a (PMC6053953; doi:10.1039/c8sc01379a)
Supplement: Supplementary file 1 [file SC-009-C8SC01379A-s001.pdf]

Electronic Supplementary Material (ESI) for Chemical Science.
This journal is © The Royal Society of Chemistry 2018

Electronic Supplementary Information

Self-assembly of toroidal proteins explored using native mass spectrometry

N. Amy Yewdall[a,b], Timothy M. Allison[c], F. Grant Pearce[a], Carol V. Robinson[c†], Juliet A. Gerrard[b,d†]

[a] School of Biological Sciences and School of Chemical Sciences, University of Auckland, Auckland 1010, New Zealand

[b] Biomolecular Interaction Centre and School of Biological Sciences, University of Canterbury, Christchurch 8140, New Zealand

[c] Department of Chemistry, University of Oxford, Oxford OX1 5QY, United Kingdom

[d] MacDiarmid Institute for Advanced Materials and Nanotechnology, Victoria University, Wellington 6140, New Zealand

[†] Corresponding authors

Supplementary methods

Figures involving protein crystal structure representations were made using PyMOL. The assessment of surface area of protein-protein interactions were performed using PDBePISA on the human peroxiredoxin 3 crystal structure (PDB: 5JCG).

Table S1 Comparison of theoretical and experimental molecular weights of cleaved peroxiredoxin at pH 4.0

Number of rings	Theoretical MW (Da)	Experimental MW (Da)
1	266042	266718 ± 32
2	532085	533746 ±113
3	798127	800319 ± 86
4	1064170	1066791 ± 80
5	1330212	1333927 ± 109
6	1596254	1600556 ± 148

Table S2 Comparison of theoretical and experimental molecular weights of His_6-tagged peroxiredoxin at pH 4.0

Number of rings	Theoretical MW (Da)	Experimental MW (Da)
1	303872	305558 ± 83
2	607745	600836 ± 115* and 611046 ± 34
3	911617	916055 ± 97

* indicates MW for sample mass at 0 hours

Table S3 Rotor speed and wavelengths used for AUC experiments

Figure number	Sample name	Rotor speed (rpm)	Wavelength (nm)
S1	Cleaved peroxiredoxin, pH 8.0	42000	285
4B	Cleaved peroxiredoxin, pH 4.0	30000	285
5B	His_6-tagged peroxiredoxin, pH 4.0	24000	280

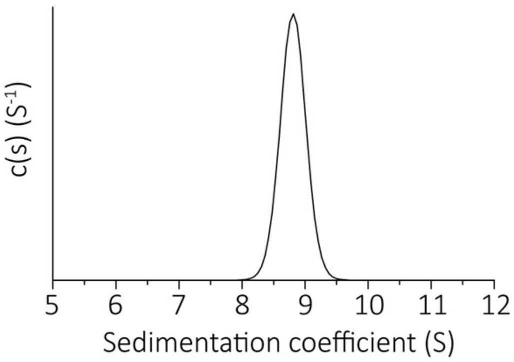

Figure S1: AUC of cleaved human peroxiredoxin 3 in 100 mM ammonium acetate at pH 8.0

The AUC experiment was conducted using purified cleaved protein (20 µM) in 100 mM ammonium acetate. The protein retains its dodecameric oligomeric state with a sedimentation coefficient of 8.8 S. The frictional ratio (f/f_0) was 1.63.

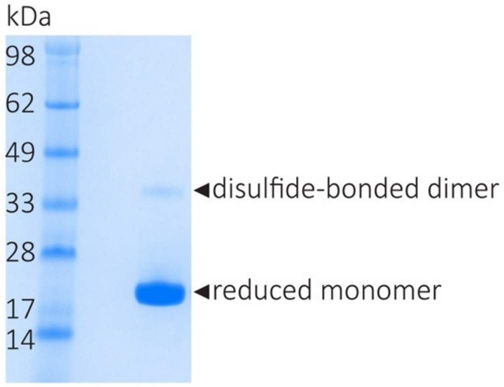

Figure S2: Non-reducing SDS-PAGE of human peroxiredoxin 3

Peroxiredoxin proteins were diluted either 1:10 or 1:15 with distilled water and immediately buffer exchanged into 100 mM ammonium acetate, pH 8.0. Loading buffer, without reducing agents, was added to the protein and the samples were run on a non-reducing SDS-PAGE. The majority of the protein population appear as reduced monomers (~22 kDa), therefore the curious CID dissociation pathway via 2-mer dissociation is unlikely to be a result of disulfide-bonded dimers.

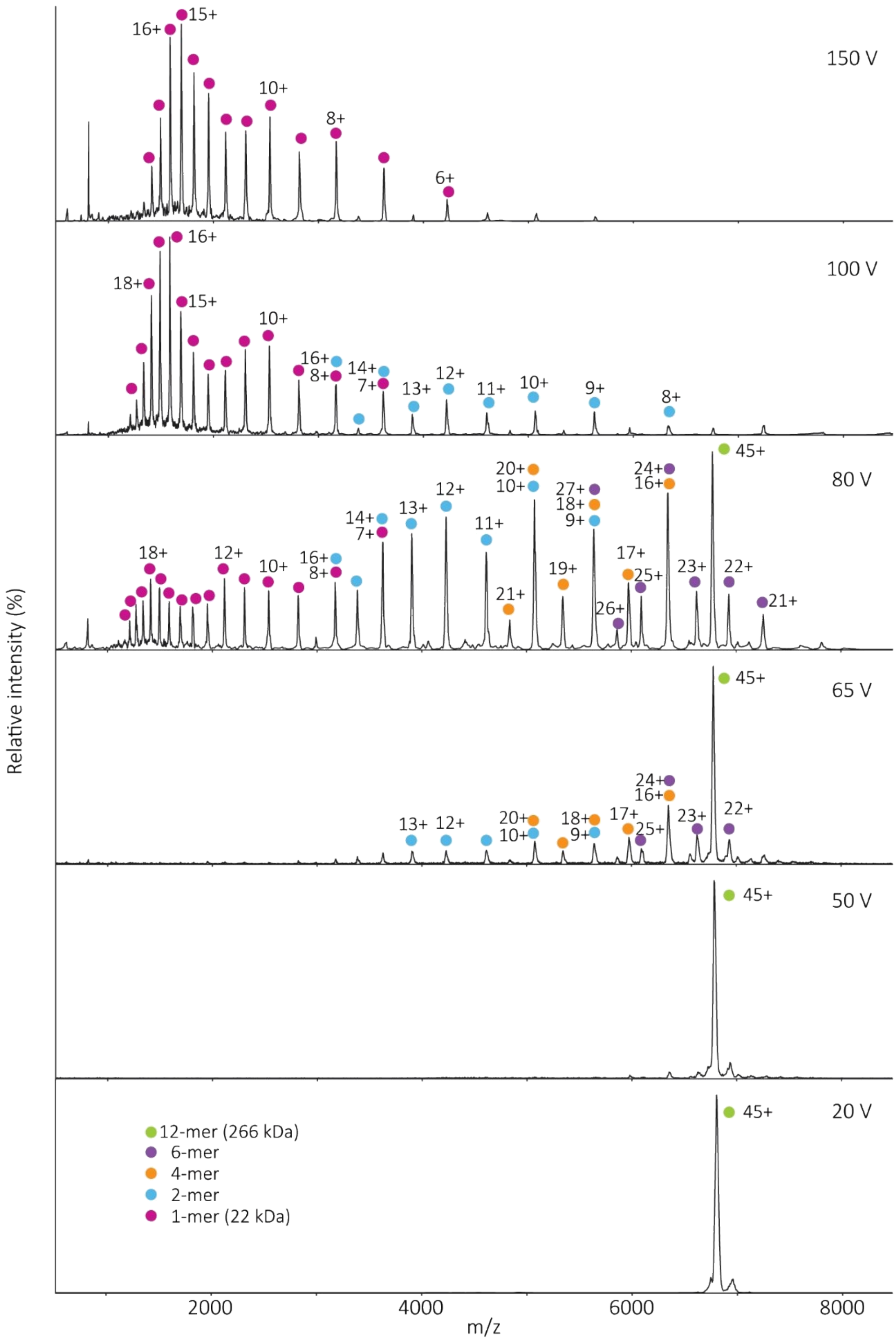

Figure S3: CID graph of His$_6$-tagged peroxiredoxin 3

MS/MS spectra recorded at different trap CE voltages (indicated on right) follows the dissociation of the isolated 6770 m/z (45+) peak of His$_6$-tagged protein. Peaks were assigned to each charge state series of the oligomer as indicated in legend below. The dissociation pathway for His$_6$-tagged proteins is similar to that of cleaved peroxiredoxin (**Fig 7**), with 6-mers being the preferred dissociation option at lower energies.
